# Computational Study of Gas-Phase Reactions to Produce Sulfite Esters: A New Class of Sulfur Containing Molecules in the Atmosphere?

**DOI:** 10.1021/acs.jpclett.6c02217

**Published:** 2026-10-11

**Authors:** Christopher David Daub, Avinash Kumar, Matti Rissanen

**Affiliations:** † Institute for Atmospheric and Earth System Research/Physics, Faculty of Science, University of Helsinki, P.O. Box 64, Helsinki FI-00014, Finland; ‡ Aerosol Physics Laboratory, Physics Unit, Faculty of Engineering and Natural Sciences, 7840 Tampere University, Tampere 33720, Finland; ¶ Department of Chemistry, University of Helsinki, P.O. Box 55, Helsinki FI-00014, Finland

## Abstract

We use computational quantum chemistry to study gas-phase reactions of aldehydes with water and sulfur dioxide. We find that a new reaction leading to the formation of previously nearly unstudied sulfite esters is possible with a comparatively low barrier. The reaction can be catalyzed by the presence of additional hydrogen bonding molecules, including water, nitric acid, and sulfuric acid. Our calculations also show that the binding energy between nitrate ions and sulfite esters should be high enough to enable their detection by nitrate ion chemical ionization mass spectrometry (CIMS). Under certain conditions, such as at the surface of acid–base clusters, the formation of these sulfite esters should be competitive with that of organosulfates and sulfinic acids. These findings suggest that sulfite esters may represent a new class of molecules that can contribute to new particle formation (NPF) and the subsequent generation of atmospheric aerosol.

## Graphical abstract

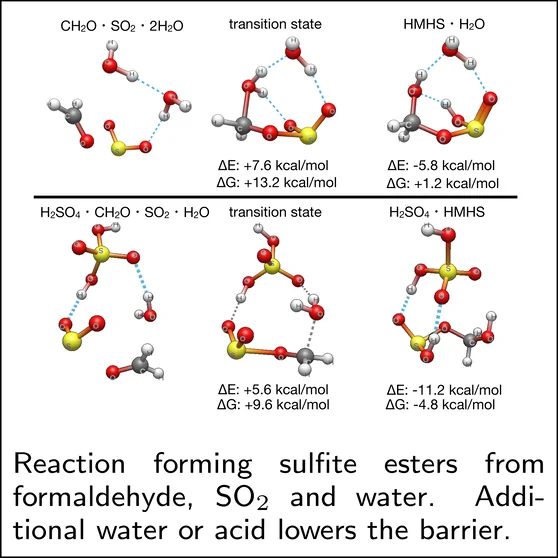

Sulfur compounds are important contributors to atmospheric chemistry. The most important and well-studied of these is sulfuric acid, arising from the water-catalyzed hydrolysis of sulfur trioxide.^1–4^ The sulfur trioxide in this reaction is formed from oxidation of sulfur dioxide (SO_2_),^5^ which is emitted in high concentrations from anthropogenic sources, as well as by volcanic activity.^6^

Although the importance of sulfuric acid for new particle formation (NPF) and hence cloud formation should not be understated, a variety of other sulfur chemistries are important in the atmosphere.^7–17^ Owing to the six *d*-orbitals in the valence shell of sulfur, it can be present in oxidation states ranging from −2 in sulfides (e.g., H_2_S, (CH_3_)_2_S), to +4 in sulfites and sulfonic acids (e.g., H_2_SO_3_, SO_2_) and +6 in sulfates (e.g., H_2_SO_4_, SO_3_). In experimental and theoretical work, we have recently examined gas-phase reactions leading to organosulfates such as formic sulfuric anhydride^18,19^ and iodous sulfuric anhydride.^20^ Besides being able to observe the formation of these compounds in a flow-tube reactor, we were able to detect the presence of these compounds in the atmosphere for the first time.^20^

Aldehydes are relatively common atmospheric molecules. Photolysis of formaldehyde to form a HCO radical followed by reactions with O_2_ produces HO_2_ radicals, which indirectly leads to it being a key source of important OH radicals in polluted conditions.^21,22^ Conversion of formaldehyde into methanediol in the gas phase, both alone and catalyzed by sulfuric acid, has been examined with quantum chemistry,^23–25^ including its contribution to NPF.^25^ The catalyzed hydrolysis reactions of acetaldehyde^26^ and glycolaldehyde^27^ have also been studied with quantum chemistry. Oxidation of acetaldehyde and other larger aldehydes can lead to the formation of acyl peroxy radicals, which can further react to form highly oxygenated organic molecules (HOMs) which play a key role in the growth of atmospheric aerosols.^28–31^ In droplets, formaldehyde converts into methanediol and then into formic acid.^32^ In the presence of SO_2_, aqueous formaldehyde can also form hydroxymethanesulfonate (HMS)^14^ and hydroxymethanesulfonic acid (HMSA).^8^

Sulfite esters,^33^ also known as organosulfites, with the general chemical formula (RO)­(R′O)­S=O are possible products of reactions between sulfur-containing molecules such as SO_2_, aldehydes, and water. They are comparable to more well-known molecules such as sulfonic acids and organosulfates, but with a different bonding pattern. For example, hydroxymethyl hydrogen sulfite (HMHS, (HOCH_2_O)­(HO)­S=O)) has a corresponding sulfonic acid with the same atomic composition, HMSA, but with a C–O bond formed instead of a C–S bond, while methyl hydrogen sulfate (MHS) is the corresponding organosulfate. To date, investigations of sulfite esters in an atmospheric context have been limited to one theoretical study of reactions between formaldehyde and bisulfite ions (HSO_3_^–^) or sulfurous acid (H_2_SO_3_) to form hydroxymethyl sulfite ions or HMHS, respectively.^34^ It was found that these reactions had low barriers, suggesting that HMHS could be formed in aerosol droplets where bisulfite ions or sulfurous acid was present, and the reaction could be competitive with the more well-studied formation of HMSA in aerosol droplets containing formaldehyde.^8^

In this study, we use quantum chemistry to examine gas-phase reactions of simple aldehydes (formaldehyde and acetaldehyde) with SO_2_ taking place in the presence of 1 to 3 water molecules or with atmospheric acids (HNO_3_ and H_2_SO_4_) in addition to a water molecule. We find that reactions with a surprisingly low barrier are possible, which lead to the formation of sulfite esters, heretofore almost completely unexamined in an atmospheric context. Although we find that SO_2_ does not bind strongly to other molecules in the gas phase, in some atmospheric environments, it can accumulate in sufficient quantity for sulfite ester formation to occur. If HMHS is indeed being formed in the atmosphere, our calculation of its binding energy with a nitrate anion supports the view that we ought to be able to detect this new species with the aid of carefully controlled atmospheric-pressure flow-tube experiments coupled with mass spectrometry for molecular detection.

We used ORCA versions 6.0.1 and 6.1.1 ^35,36^ for all of the quantum chemical computations reported in this work. Optimizations and other calculations involving gradient calculations were performed with the ωB97-X density functional theory (DFT) functional^37–39^ and the aug-cc-pVTZ basis set^40^ for all atoms besides sulfur. For sulfur, we used the aug-cc-pVT­(+d)­Z basis set since it has been shown that the inclusion of extra *d* functions is required to ensure that reactions involving the formation or breaking of S–X bonds are described accurately.^11,41,42^ All of the DFT calculations included the D4 dispersion correction.^43–45^

The DFT results were complemented in some cases with canonical CCSD­(T1)-F12/RI and/or DLPNO-CCSD­(T1)-F12 ^46–50^ single-point energies, using cc-pVTZ-F12 basis sets and cc-pVQZ or aug-cc-pVQZ auxiliary basis sets. These were mainly used to more accurately determine the reaction energetics for sulfite ester formation, as well as for quantifying the possible importance of multireference effects. For more accurately calculating binding energies to the nitrate ion, we performed single-point DLPNO-CCSD­(T) calculations at the DFT-based geometries. Coupled-cluster calculations of relative energies for systems like those studied here can generally be considered accurate to within 1–2 kcal mol^–1^.^51^

Conformational sampling is essential to ensure that we study the most energetically favorable molecular geometries. This is especially important in our case, since we study clusters with many local minima. We do conformational sampling using the so-called Global Optimizer AlgoriThm (GOAT),^52^ with energies calculated with the GFN1-XTB semiempirical method,^53,54^ followed by reoptimization using DFT as just described to find the most energetically favorable conformations. The GOAT method was reliable for determining the global minima of all of the single molecules mentioned in our study with only a small number of relevant local minima for each molecule. For clusters, however, especially those with more than two molecules, even the new GOAT methodology should not be assumed to have completely explored the conformational space or to have unambiguously determined the global minima in all cases.

To find transition states and measure reaction barriers, we used nudged elastic band (NEB) calculations.^55^ NEB calculations can find transition states starting only from knowledge of the optimized reactant and product minima by building an initial path of geometries connecting the minima and gradually optimizing along this pathway to find a minimum energy path, followed by a standard transition state (TS) optimization at the highest point of the path. This method was ideal for our situation since we had little a priori information about what the unknown reaction pathway might be.

NEB calculations beginning from the best reactant conformations and ending with the best product conformations were generally successful at finding transition states. Next, we calculated the intrinsic reaction coordinate (IRC)^56^ starting from each transition state, followed by another optimization at each end of the IRC to determine the actual reactant and product configurations being linked by the transition state found by the NEB-TS calculation. In most cases, these differ somewhat from the global minimum reactant and product configurations.

In the top part of Figure 1 we show the minimum energy configuration for HMHS. For comparison, some other related molecules,^13,57,58^ such as sulfinic acids and organosulfates, and the prereactive clusters are shown as well. A similar graphic for 1-hydroxyethyl hydrogen sulfite (HEHS) and other related molecules that could result from reactions with acetaldehyde can be found in the Supporting Information (Figure S1).

**Figure 1:**
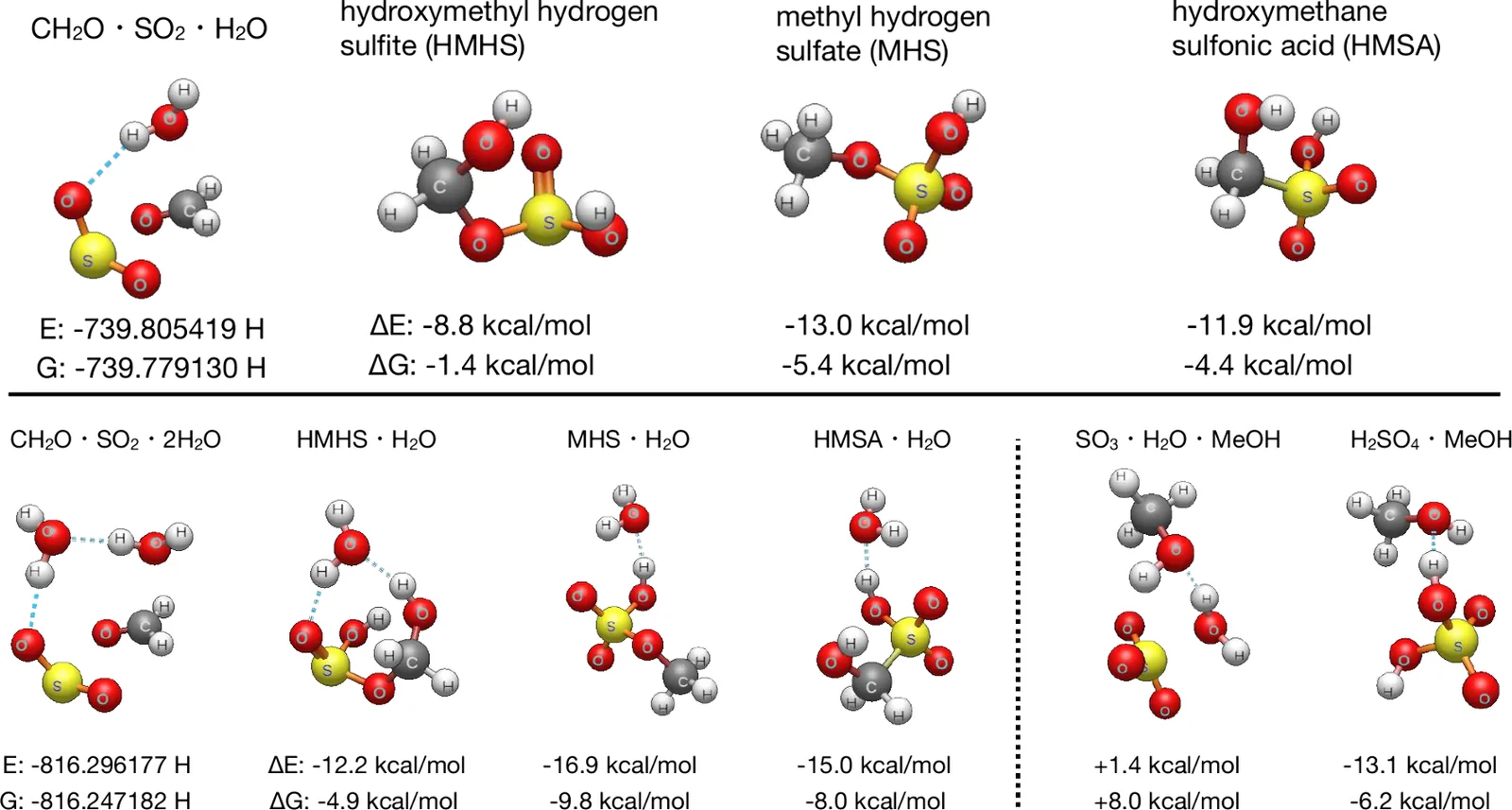
(Top) Global minima for hydroxymethyl hydrogen sulfite (HMHS), as well as the corresponding sulfonic acids and organosulfates, and the relevant prereactive clusters. (Bottom) Global minima for complexes of H_2_O with HMHS, HMSA, and organosulfates under consideration. Relative energies are in kcal mol^–1^ relative to the absolute electronic energy or Gibbs free energy of the corresponding prereactive clusters given in hartrees.

The lower part of Figure 1 shows the minimum energy configurations for our molecules of interest, complexed with one water molecule. Some other relevant configurations of the same number of atoms which have been studied before are included,^13,57,58^ such as the complex of methanol with sulfuric acid and the complex of methanol with SO_3_ and one water molecule. The sulfite ester configurations are close in energy to other more well-studied conformations of these atoms. For example, the difference in Gibbs free energy for the HMHS·H_2_O cluster is Δ*G* = 1.4 and 5.0 kcal mol^–1^ compared to the sulfuric acid-methanol cluster and the MHS·H_2_O cluster, respectively.

We have also determined some optimized geometries and binding energies for clusters of these molecules with the nitrate anion (NO_3_^–^), shown in Figure 2. Nitrate ion chemical ionization mass spectrometry (CIMS) is used routinely for sulfuric acid detection and has been shown to work well to detect sulfuric acid anhydride and other organosulfates,^20,59–61^ so it is also a good choice for attempts to detect sulfite esters. The binding free energy of the HMHS-nitrate complex is found to be approximately −22 kcal mol^–1^, which should enable its highly sensitive detection in nitrate ion CIMS since its binding free energy exceeds the binding free energy of the HNO_3_·NO_3_^–^ cluster of approximately −20 kcal mol^–1^.^62,63^ However, as also shown, other related molecules with the same chemical composition also bind strongly to nitrate, which could make it difficult to distinguish them without the use of sophisticated MS/MS techniques.

**Figure 2:**
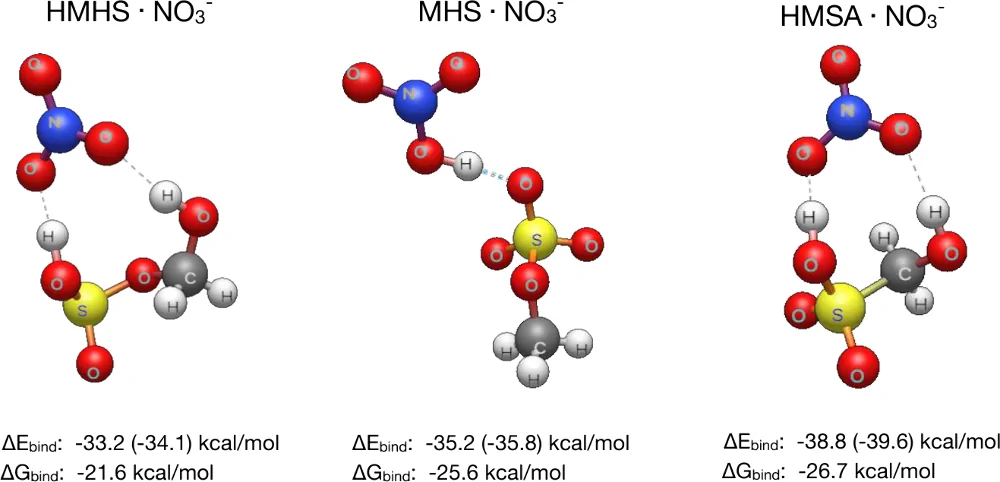
Geometries and binding energies of the global minima for complexes of a nitrate anion with HMHS, MHS, and HMSA. Values in parentheses are from single-point calculations with DLPNO-CCSD­(T), with all other values from DFT calculations as described in the text.

We now turn our focus to the calculation of transition states and barrier heights we found for the previously unstudied reaction(s) to produce sulfite esters of interest. In Table 1 we summarize our findings for the barrier heights for the formation of sulfite esters. Note that in most cases, the minima connected by the IRC calculation are different from the global minima determined by conformational sampling, so the barrier heights compared to the global minima may be ∼1–3 kcal mol^–1^ higher in some cases. As these calculations are the most important results in this study, we have also provided all of the relevant input and output files and configurations in the Supporting Information.

**Table 1:** Barrier Heights for the Formation of Sulfite Esters from the Prereactive Complexes of Aldehyde·SO_2_·*n*H_2_O^a^

Prereactive system	Δ*E*_barrier_ (kcal mol^–1^)	Δ*G*_barrier_ (kcal mol^–1^)
CH_2_O·SO_2_·H_2_O	12.8(15.9)	18.4(21.5)
CH_2_O·SO_2_·2H_2_O	4.9(7.6)	10.5(13.2)
CH_2_O·SO_2_·3H_2_O	10.0	13.7
CH_3_CHO·SO_2_·H_2_O	16.8	21.9
CH_3_CHO·SO_2_·2H_2_O	7.0	12.9
CH_3_CHO·SO_2_·3H_2_O	12.4	16.9

a Values in parentheses include an adjustment based on DLPNO-CCSD­(T1)-F12 single-point energies. The barrier height is relative to the energy of each prereactive complex. Note that the geometries (and energies) of the prereactive complexes and the products may differ from the global minima shown in Figure 1 and Figure S1.

The energy minima and transition state energies were further refined by doing coupled-cluster single-point energy calculations. This generally led to an increase of ∼2–3 kcal mol^–1^ in the barrier height compared with the DFT results. These calculations also allowed us to monitor the possible impact of multireference effects, since these have been shown to be important in other reactions involving the formation of new sulfur–oxygen bonds in reactions involving sulfur peroxy radicals.^10,11^ Our results show that the *T*_1_ diagnostic consistently remains below the threshold of 0.02, usually considered to indicate that multireference effects may be important. We also did some calculations of the new *S* diagnostic, which is a more direct measurement of how important multireference effects may be.^64^ This calculation was limited to our smallest system (shown in Figure 3A), since it requires canonical coupled-cluster methods. The value of the *S* diagnostic is 0.65, well under values of *S* ∼ 1 which may indicate that multireference methods must be used. A table of the coupled-cluster results and the multireference diagnostics can be found in Table S1 of the Supporting Information.

**Figure 3:**
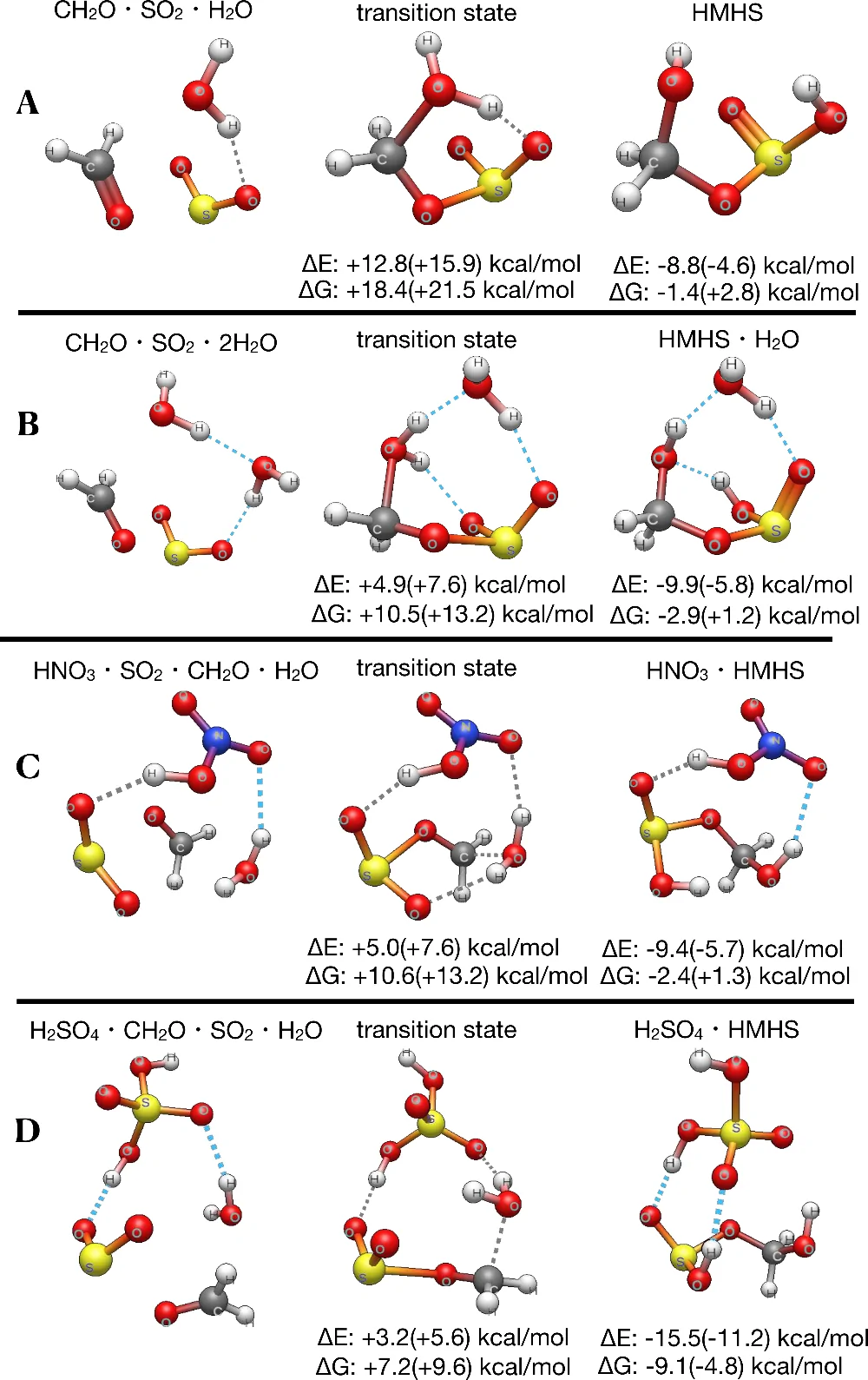
Reactant, transition state, and product configurations for the reaction of formaldehyde, SO_2_, and water to form HMHS, with (A) one or (B) two water molecules in the prereactive cluster, and with one water and (C) HNO_3_ or (D) H_2_SO_4_ in the prereactive cluster. Energies in parentheses have been adjusted based on DLPNO-CCSD­(T1)-F12 or canonical CCSD­(T1)-F12/RI single-point energies.

The results show that when the prereactive complex includes two water molecules, the additional water molecule catalyzes the reaction, lowering the barriers for sulfite ester formation significantly: from 12.8 to 4.9 kcal mol^–1^ (15.9 to 7.6 from coupled-cluster calculations) for formaldehyde and from 16.8 to 7.0 kcal mol^–1^ for acetaldehyde. The transition states for the sulfite ester formation from formaldehyde including one and two water molecules, as well as the reactant and product states revealed by the IRC calculation, are shown in Figure 3A,B. The reaction involves a proton transfer, with one water molecule binding to the carbon before transferring one hydrogen to a sulfite oxygen, while simultaneously the formaldehyde oxygen forms an O–S bond.

Although one additional water molecule lowers the barrier, it does not appear to directly participate in the reaction. Furthermore, when more additional water molecules are present, the barrier does not decrease further, although it is difficult to be certain about this. As many possible configurations are available, especially when more water molecules are included, it is possible that some configurations would ideally promote the reaction, perhaps by a more complex double proton transfer as seen in, for example, the catalyzed SO_3_ hydrolysis reaction to form sulfuric acid.^2^ However, we were not successful in sampling them in this study.

The simpler but related reaction between water and SO_2_ catalyzed by water has been studied previously.^7^ The uncatalyzed reaction to form H_2_SO_3_ was found to have an enthalpic barrier of 34 kcal mol^–1^, reduced to 20 kcal mol^–1^ when catalyzed by an additional H_2_O. In related calculations of the CH_2_O·SO_2_·2H_2_O cluster not further discussed here, we found a transition state with a barrier of 28 kcal mol^–1^ for the formation of H_2_SO_3_·CH_2_O·H_2_O, a considerably higher barrier than the one we report here for sulfite ester formation. Further, the formation of HMSA·H_2_O from H_2_SO_3_·CH_2_O·H_2_O (also not shown here) requires overcoming an energetic barrier of 24 kcal mol^–1^. While these reactions presumably can happen in the aqueous phase with considerably lower barriers,^8^ based on our results it seems clear that the HMHS sulfite ester should be the most likely possible reaction occurring from the CH_2_O·SO_2_·2H_2_O cluster.

Other hydrogen bonding molecules can also act as catalysts for the sulfite ester formation instead of water. To investigate this, we replaced the catalyst water molecule with nitric acid (HNO_3_) and sulfuric acid (H_2_SO_4_). The reactant, transition state, and products for these reactions are shown along with the water-catalyzed reactions in Figure 3C,D, as well as the energies relative to the energies of the reactant complexes. Nitric acid is nearly as effective as water as a catalyst for this reaction, while sulfuric acid is even more effective, lowering Δ*E*_barrier_ to 3.2 kcal mol^–1^ and Δ*G*_barrier_ to 7.2 kcal mol^–1^. Methanediol formation from the hydrolysis of formaldehyde catalyzed by sulfuric acid has been found to have an energetic barrier relative to the prereactive complex of ∼6.1 kcal mol^–1^,^23^ while a reaction between formaldehyde and sulfuric acid to form hydroxymethyl hydrogen sulfate could also occur in this system; however, this reaction has been found to have an energetic barrier relative to the prereactive complex of ∼10 kcal mol^–1^.^23^ Overall, our results suggest that the most likely reaction which could result from a H_2_SO_4_·H_2_O·SO_2_·CH_2_O cluster would be the HMHS sulfite ester.

Although water, SO_2_, and CH_2_O are relatively plentiful atmospheric molecules, if the reactant clusters shown in Figure 3 do not form under typical conditions, then it is unlikely that sulfite esters would form via this exact gas-phase reaction mechanism in the atmosphere in significant quantities.

In Table 2 we show our calculations of binding energies for the relevant 2-, 3-, and 4-molecule clusters involved in possible reactant cluster formation. It should be noted that we did not thoroughly explore the conformational space of the clusters, so it is possible that somewhat lower global minima exist. Nevertheless, it seems clear that the reactant clusters are not sufficiently strongly bound to accumulate in significant quantities. While further kinetic calculations to determine the rates of formation of all of the clusters and then the sulfite ester product could have been performed, since the results would have almost certainly indicated very low rates, we have decided not to include them in this short Letter.

**Table 2:** Binding Energies for 2-, 3-, and 4-Molecule Clusters Relevant to Possible Sulfite Ester Formation^a^

	Δ*E*_bind_	Δ*G*_bind_
Bimolecular		
SO_2_·CH_2_O	–6.4	3.8
2H_2_O	–4.9	2.7
H_2_O·SO_2_	–5.2	3.2
H_2_O·CH_2_O	–5.3	3.2
HNO_3_·H_2_O	–10.4	0.1
HNO_3_·SO_2_	–3.5	6.4
HNO_3_·CH_2_O	–10.5	0.7
H_2_SO_4_·H_2_O	–12.6	–1.5
H_2_SO_4_·SO_2_	–9.1	2.5
H_2_SO_4_·CH_2_O	–12.6	–1.1
Trimolecular		
H_2_O·SO_2_·CH_2_O	–13.2	7.8
2H_2_O·SO_2_	–14.6	5.9
2H_2_O·CH_2_O	–8.5	2.5
HNO_3_·SO_2_·CH_2_O	–16.4	6.4
HNO_3_·H_2_O·SO_2_	–14.6	6.0
HNO_3_·H_2_O·CH_2_O	–16.0	3.7
H_2_SO_4_·SO_2_·CH_2_O	–21.9	2.5
H_2_SO_4_·H_2_O·SO_2_	–22.5	0.9
H_2_SO_4_·H_2_O·CH_2_O	–22.0	0.8
4 Molecules		
2H_2_O·SO_2_·CH_2_O	–11.9	20.9
HNO_3_·H_2_O·SO_2_·CH_2_O	–21.7	12.3
H_2_SO_4_·H_2_O·SO_2_·CH_2_O	–24.4	11.0

a All energies are in kcal mol^–1^ and are relative to the energies of the isolated constituent molecules.

Clusters of SO_3_ with water and other small atmospheric molecules are known to play an important role, especially in the formation of atmospheric sulfuric acid. The comparatively small binding energy of SO_2_ with the other molecules compared with SO_3_ is likely to be the main reason for the difference in overall cluster formation probability and lifetimes and hence in the importance of reactions occurring within those small clusters.

Nevertheless, there are atmospheric environments where the formation of sulfite esters directly from SO_2_ may still occur; therefore, we present our results mainly as a model for what could happen in these environments. For example, it is possible that SO_2_, formaldehyde, and water molecules could bind to the surface of an acid–base cluster including sulfuric acid, followed by the sulfuric-acid-catalyzed formation of HMHS as depicted in Figure 3D. It has recently been speculated that the formation of accretion products from smaller HOMs could be accelerated at the surface of an acid–base cluster in a similar way.^65^ Alternately, the water-catalyzed reaction (Figure 3B) could take place at the surface of a SO_2_ and formaldehyde-rich droplet, competing with the more well-known generation of HMSA or other S­(IV) species in atmospheric droplets,^8,14^ as well as offering an alternative to the predicted solution-phase route for generating sulfite esters.^34^

In conclusion, we have used quantum computational chemistry methods to study the reaction of SO_2_ with aldehydes, water, and atmospheric acids in the gas phase. We have uncovered a previously unexamined reaction to form sulfite esters. When two water molecules are present in the initial cluster, the reaction of formaldehyde with SO_2_ to form HMHS has a low barrier relative to the prereactive complex (Δ*E*_barrier_ ∼ 7.6 kcal mol^–1^, Δ*G*_barrier_ ∼ 13.2 kcal mol^–1^), with an even lower barrier possible when the catalyst water molecule is replaced with sulfuric acid. Our study suggests that the formation of hydroxymethyl hydrogen sulfite (HMHS) in the atmosphere or in laboratory experiments should be possible under certain conditions such as at the surface of an acid–base particle. Furthermore, the binding energy of HMHS with a nitrate anion ought to be high enough to enable its detection by nitrate-CIMS instruments with very high sensitivity.

To the best of our knowledge, although sulfite ester formation in aerosol particles has been studied theoretically,^34^ our putative new reaction to form sulfite esters in the gas phase or at an interface has never been studied for any kind of atmospheric relevance. It is possible that HMHS has actually been seen during measurement campaigns, but due to the limitations of mass spectrometric methods it was mistaken for other molecules with the same mass (e.g., HMSA or MHS) That being said, it may be challenging to seek out the formation of sulfite esters in flow-tube experiments. Although water or other hydrogen bonding donor molecules play a crucial role in this reaction to form the sulfite ester, we know that in aqueous solutions of SO_2_ and aldehydes, the formation of alkyl thiols and/or alkylsulfonic acids is observed.^8^ It may prove difficult to create initial conditions where the prereactive clusters that can lead to sulfite ester formation have an appreciable population or to observe sulfite ester formation on the surface of an aerosol particle. It will also be difficult to unambiguously identify HMHS rather than HMSA or MHS without the use of sophisticated MS/MS methods. These factors may also explain, in part, why sulfite esters have not been previously observed in field experiments. Nevertheless, we intend to pursue this goal in our future work in addition to continuing to explore more exotic sulfur chemistries in the atmosphere.

## Supplementary Material





## Notes

ZIP archive containing input and output files and configurations required to make Figure 3 (ZIP)
Products of acetaldehyde reactions with SO_2_ and water (analogous to Figure 1) and coupled-cluster results, including multireference diagnostics (PDF)
