# Supplementary material for: Computational Study of Gas-Phase Reactions to Produce Sulfite Esters: A New Class of Sulfur Containing Molecules in the Atmosphere?
Source: J Phys Chem Lett. 2026 Oct 11;17(42):12164–70. doi: 10.1021/acs.jpclett.6c02217 (PMC13700091; doi:10.1021/acs.jpclett.6c02217)
Supplement: Supplementary file 1 [file jz6c02217_si_002.pdf]

Supporting Information: Computational Study of Gas-Phase Reactions to Produce Sulfite Esters: A New Class of Sulfur Containing Molecules in the Atmosphere?

Christopher David Daub,[*,†,‡] Avinash Kumar,[‡,¶] and Matti Rissanen[*,‡,¶]

†*Institute for Atmospheric and Earth System Research/Physics, Faculty of Science, University of Helsinki, P.O. Box 64, Helsinki FI-00014, Finland*

‡*Aerosol Physics Laboratory, Physics Unit, Faculty of Engineering and Natural Sciences, Tampere University, Tampere 33720, Finland*

¶*Department of Chemistry, University of Helsinki, P.O. Box 55, Helsinki FI-00014, Finland*

E-mail: christopher.daub@helsinki.fi; matti.rissanen@tuni.fi

Products of acetaldehyde, SO_2 and water reactions

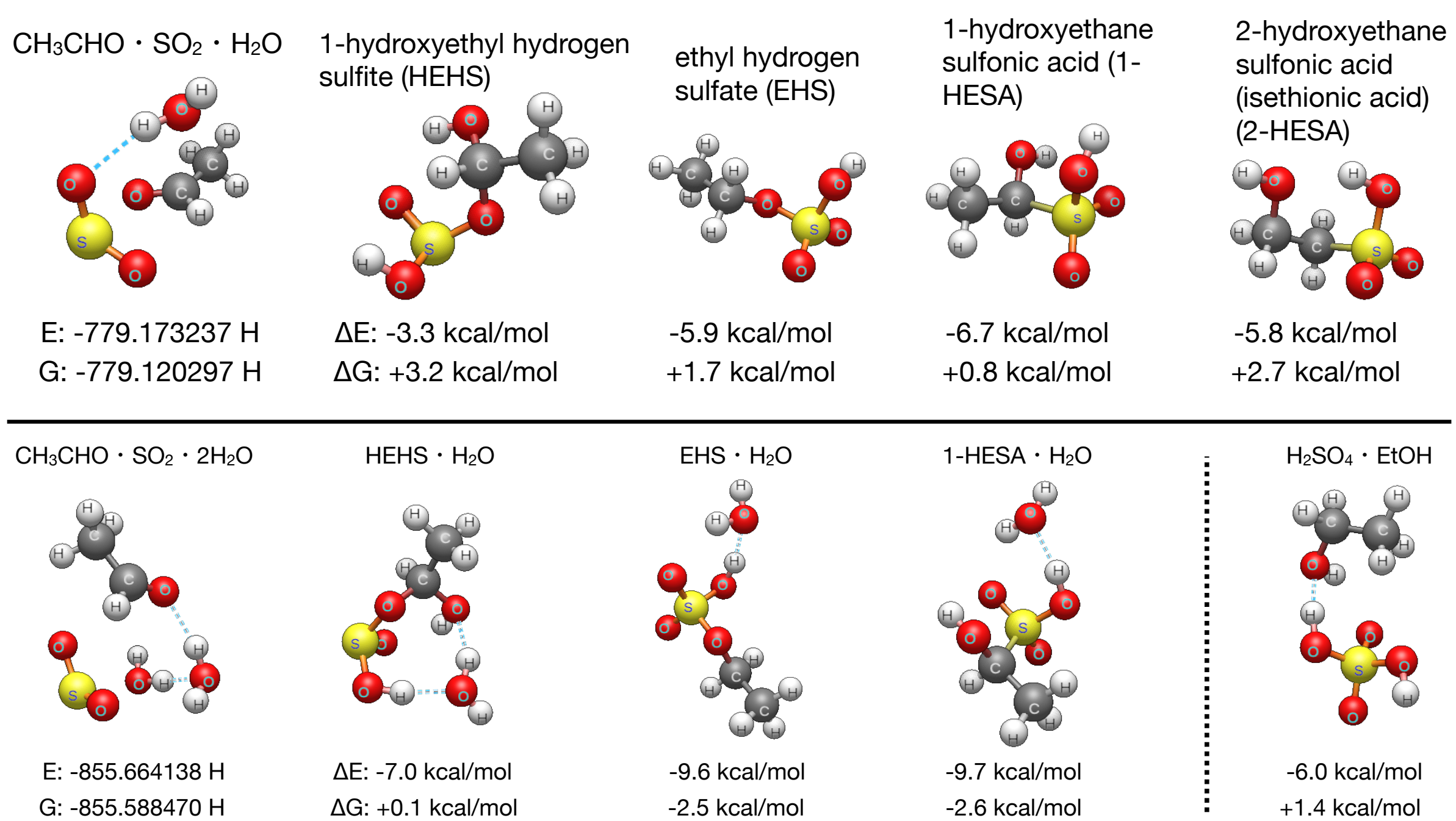

Figure S1: (Top) Global minima for 1-hydroxyethyl hydrogen sulfite (HEHS), as well as the corresponding sulfonic acids and organosulfates, and the relevant prereactive clusters. (Bottom) Global minima for complexes of one water molecule with HEHS, ethylsulfonic acids and organosulfates formed from reactions with acetaldehyde. Energies are in kcal/mol relative to the absolute electronic energy or Gibbs free energy of the corresponding prereactive clusters given in Hartrees. All calculations are DFT calculations as described in the main text.

Coupled-Cluster calculations including multi-reference diagnostics for sulfite ester forming reactions

Table S1: Single point energies, ΔE_{PRC} relative to the prereactive complex, T_1 and S diagnostics for all of the reactions/configurations shown in Figure 3 of the main text. "DLPNO" refers to a DLPNO-CCSD(T1)-F12 calculation with the cc-pVTZ-F12 basis set and cc-pVQZ or aug-cc-pVQZ auxiliary basis sets. "Canonical" refers to a canonical CCSD(T1)-F12/RI calculation with the same basis sets.

System	Method	E/Hartrees	ΔE_{PRC} / kcal mol^{-1}	T_1	S_{diag}
$H_2O \cdot SO_2 \cdot CH_2O$	DLPNO	-738.841610	0	0.018	
TS		-738.816008	16.1	0.018	
HMHS		-738.848684	-4.4	0.015	
$H_2O \cdot SO_2 \cdot CH_2O$	canonical	-738.848407	0	0.018	0.63
TS		-738.823070	15.9	0.019	0.64
HMHS		-738.855714	-4.6	0.016	0.58
$2H_2O \cdot SO_2 \cdot CH_2O$	DLPNO	-815.221375	0	0.017	
TS		-815.209260	7.6	0.017	
HMHS $\cdot H_2O$		-815.230659	-5.8	0.014	
$HNO_3 \cdot H_2O \cdot SO_2 \cdot CH_2O$	DLPNO	-1019.486927	0	0.018	
TS		-1019.474773	7.6	0.018	
HMHS $\cdot HNO_3$		-1019.495962	-5.7	0.017	
$H_2SO_4 \cdot H_2O \cdot SO_2 \cdot CH_2O$	DLPNO	-1438.474344	0	0.016	
TS		-1438.465434	5.6	0.017	
HMHS $\cdot H_2SO_4$		-1438.492197	-11.2	0.015	
